# Adipose-derived mesenchymal stem cells attenuate dialysis-induced peritoneal fibrosis by modulating macrophage polarization via interleukin-6

**DOI:** 10.1186/s13287-021-02270-4

**Published:** 2021-03-19

**Authors:** Chih-Yu Yang, Pu-Yuan Chang, Jun-Yi Chen, Bo-Sheng Wu, An-Hang Yang, Oscar Kuang-Sheng Lee

**Affiliations:** 1 Institute of Clinical Medicine, School of Medicine, National Yang Ming Chiao Tung University, 2F, Shou-Ren Bldg., No.155, Sec.2, Li-Nong St., Beitou Dist, Taipei, 11221 Taiwan; 2 Faculty of Medicine, School of Medicine, National Yang Ming Chiao Tung University, Taipei, 11221 Taiwan; 3 Division of Nephrology, Department of Medicine, Taipei Veterans General Hospital, Taipei, 11217 Taiwan; 4 Stem Cell Research Center, National Yang Ming Chiao Tung University, Taipei, 11221 Taiwan; 5 Center for Intelligent Drug Systems and Smart Bio-devices (IDS2B), Hsinchu, 30010 Taiwan; 6 Department of Pathology, Taipei Veterans General Hospital, Taipei, 11217 Taiwan; 7 Department of Orthopedics, China Medical University Hospital, Taichung, 40447 Taiwan

**Keywords:** Adipose-derived mesenchymal stem cell, Peritoneal dialysis, Peritoneal fibrosis, Macrophage, Interleukin-6

## Abstract

**Background:**

Life-long peritoneal dialysis (PD) as a renal replacement therapy is limited by peritoneal fibrosis. Previous studies showed immunomodulatory and antifibrotic effects of adipose-derived mesenchymal stem cells (ADSCs) on peritoneal fibrosis. However, the role of the peritoneal macrophage in this process remains uninvestigated.

**Methods:**

We examined the therapeutic effects of ADSC and bone marrow-derived mesenchymal stem cells (BM-MSC) in the rat model of dialysis-induced peritoneal fibrosis using methylglyoxal. In addition, treatment of macrophages with the conditioned medium of ADSC and BM-MSC was performed individually to identify the beneficial component of the stem cell secretome.

**Results:**

In the in vivo experiments, we found dialysis-induced rat peritoneal fibrosis was attenuated by both ADSC and BM-MSC. Interestingly, ADSC possessed a more prominent therapeutic effect than BM-MSC in ameliorating peritoneal membrane thickening while also upregulating epithelial cell markers in rat peritoneal tissues. The therapeutic effects of ADSC were positively associated with M2 macrophage polarization. In the in vitro experiments, we confirmed that interleukin-6 (IL-6) secreted by MSCs upon transforming growth factor-β1 stimulation promotes M2 macrophage polarization.

**Conclusions:**

In dialysis-induced peritoneal fibrosis, MSCs are situated in an inflammatory environment of TGF-β1 and secrete IL-6 to polarize macrophages into the M2 phenotype. Our findings reveal a previously unidentified role of tissue macrophage in this antifibrotic process. ADSC has the advantage of abundance and accessibility, making the application values extremely promising.

**Graphical abstract:**

In dialysis-induced peritoneal fibrosis, peritoneal mesothelial cells secrete transforming growth factor-β1 (TGF-β1) when exposed to methylglyoxal (MGO)-containing peritoneal dialysate. When situated in TGF-β1, the inflammatory environment induces mesenchymal stem cells to secrete interleukin-6 (IL-6), IL-6 polarizes macrophages into the M2 phenotype. The dominant peritoneal tissue M2 macrophages, marked by upregulated Arg-1 expression, account for the attenuation of MGO-induced dedifferentiation of peritoneal mesothelial cells to maintain epithelial integrity. 
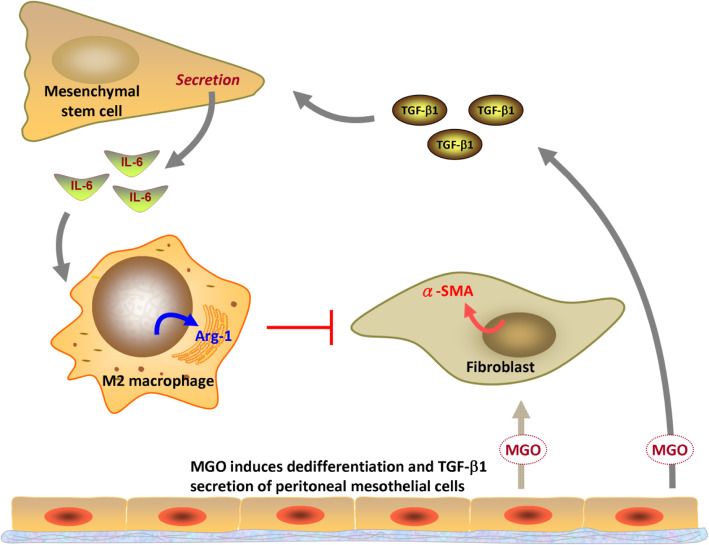

**Supplementary Information:**

The online version contains supplementary material available at 10.1186/s13287-021-02270-4.

## Introduction

Life-long peritoneal dialysis (PD) as a renal replacement therapy is limited by peritoneal fibrosis. Several studies have shown that hypertonic glucose solution is not only toxic to mesothelial cells [1, 2] but also promotes immune cell apoptosis [3]. The components of a healthy peritoneal tissue include a single layer of mesothelial cells and a submesothelial compact collagenous zone composing of fibroblasts, macrophages, vessels, and extracellular matrix [4, 5]. Peritoneal fibrosis (PF) is characterized by fibrotic changes in the peritoneal membrane, the dedifferentiation of peritoneal mesothelial cells, accumulation of submesothelial extracellular matrix, and submesothelial vasculopathy [6, 7]. TGF-β1, as a critical fibrogenic factor, is induced in peritoneal mesothelial cells by exposing them to PD dialysate containing high concentrations of glucose [8].

Emerging evidence demonstrates that inflammatory macrophages (M1) and the alternatively activated macrophages (M2) participate in the pathogenesis of various fibrotic diseases [9–11]. The M1 macrophage population participates in fibrogenesis, suggesting a requirement for inflammation in fibrosis development. In contrast, M2 macrophage secretes IL-10 and arginase-1 and exerts its anti-inflammatory properties [12, 13]. Therefore, tissue macrophage polarization is the key to regulate tissue fibrosis [14, 15].

However, therapeutic strategies targeting these pathogenic processes have not been fully developed [4]. Mesenchymal stem cell (MSC) therapies are plausible as MSCs can be isolated and propagated with ease in vitro while also with strong immunomodulatory properties [16–18]. Accumulating evidence has indicated the therapeutic potentials of MSCs in rebuilding damaged or diseased tissues as well as in the treatment of neurodegenerative disorders [19]. MSCs obtained from the bone marrow (BM-MSC) and adipose tissue (ADSC) are different in terms of their differentiation potentials, gene expression, proteomic profiles, and immunological properties [20–22].

MSCs have been shown to possess antifibrotic effects, but very few studies targeted peritoneal fibrosis [23–27]. On the other hand, ADSC facilitates chlorhexidine gluconate-induced peritoneal fibrosis repair by suppressing the dedifferentiation process of the peritoneal mesothelial cells [23]. A recent paper demonstrated that ADSC reduces leukocyte infiltration and ameliorates peritoneal fibrosis [27]. However, the role of the peritoneal macrophage in this process remains unknown.

Adipose tissue is easier to access than the bone marrow, thus a promising source for autologous cell-based therapy [28]. We aim to elucidate the therapeutic mechanisms of these two kinds of stem cells in dialysis-induced peritoneal fibrosis, with a focus on peritoneal macrophages.

## Methods

### Cell culture

Commercially available human ADSCs were purchased from Steminent Biotherapeutics Inc., Taipei, Taiwan [29, 30]. Human BM-MSCs were purchased from Cell Applications, Inc., San Diego, CA, USA [31]. Osteogenesis, chondrogenesis, and adipogenesis were confirmed in both cells by alkaline phosphatase and von Kossa stainings, type II collagen staining, and Oil Red staining. ADSC and BM-MSC were maintained in Iscovis modified Dulbecco’s medium (IMDM, Sigma-Aldrich, MO, USA) with 10% fetal bovine serum (FBS). The NR8383 rat macrophage cell line was purchased from the Bioresource Collection and Research Center, Hsinchu, Taiwan. The NR8383 culture medium consisted of Ham’s F12K medium with 15% FBS. These two media were also supplemented with 100 units/mL of penicillin, 1000 units/mL of streptomycin, and 2 mM of L-glutamine (Sigma-Aldrich, St. Louis, MO, USA). All cells were incubated at 37 °C, 5% CO_2_, and 95% relative humidity.

### The rat model of dialysis-induced peritoneal fibrosis

**Fig. 1:**
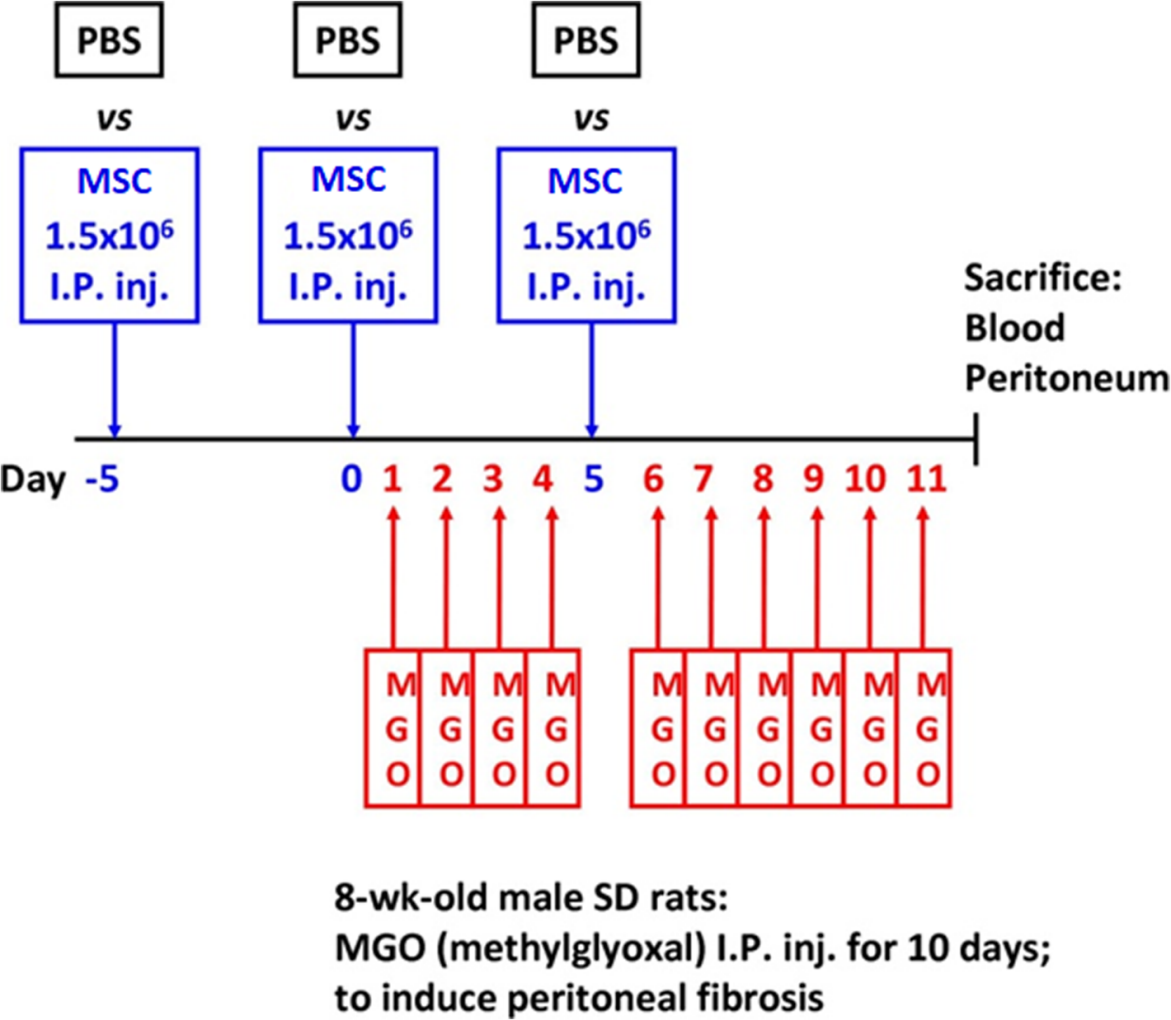
Experimental design of the peritoneal fibrosis animal model. Abbreviations: PBS phosphate buffer saline, MSC mesenchymal stem cells, I.P. inj. intraperitoneal injection, MGO methylglyoxal, SD Sprague Dawley

All animal experiments were performed following the guidelines of the Institutional Committee for Animal Experimentation of Taipei Veterans General Hospital. Male Sprague-Dawley (SD) rats were purchased from the National Laboratory Animal Center, Taipei, Taiwan. As shown in Fig. 1, we established the dialysis-induced peritoneal fibrosis by intraperitoneal injection of methylglyoxal (MGO, 6 mM) in 50 mL/kg of PDF for 10 days in 8-week-old male SD rats as previously reported [8]. PD fluid was prepared according to the literature [32, 33]. Before MGO injection, we treated the experimental rats with intraperitoneal human MSCs, including ADSC and BM-MSC. For the “MGO only” group, sterile PBS was injected into the peritoneal cavity instead. After a 10-day injection of MGO, we sacrificed the rats for peritoneal tissue and blood sample collection. The peritoneal tissues were both preserved in formalin and frozen for pathological examinations.

### Histopathological examination

At the end of the experiments, the rats were euthanized. Peritoneal tissues were taken from each rat. Peritoneal tissue was fixed in 4% formaldehyde for hematoxylin and eosin (H&E) staining according to standard protocol; samples were embedded in paraffin and cut into 4-μm-thick sections. One section from each tissue sample was stained with H&E. The H&E-stained sections were digitalized using a histological evaluation and were performed with a Panoramic MIDI digital slide scanner (3DHISTECH, Budapest, Hungary) at its highest resolution. Images were captured with Pannoramic viewer software (3DHISTECH, Budapest, Hungary).

### Masson’s trichrome staining and thickness quantification

For peritoneal thickness quantification, parietal peritoneum sized 1.0 × 0.5 cm were sampled from four separate peritoneal sites of each rat, including right ventral, left ventral, right lateral, and left lateral abdomen. For each sampling site, the peritoneal thickness was averaged from 15 evenly measured points. Tissue sections were stained with Masson’s trichrome to quantify peritoneal fibrosis. The stained sections were digitalized using a Panoramic MIDI digital slide scanner at its highest resolution. Images were captured, and peritoneal thickness was measured with Pannoramic viewer software.

### Immunohistochemical staining

We performed immunohistochemical (IHC) staining on 4-μm formalin-fixed and paraffin-embedded rat peritoneal tissue sections following routine procedures. Briefly, after incubating with primary antibody against rat cytokeratin, N-cadherin, α-smooth muscle actin (α-SMA), inducible nitric oxide synthase (iNOS), Arginine-1 (ARG-1), or human nucleoli (Table S1), all sections were labeled using a polymer-HRP staining kit (EnVision, Dako, Glostrup, Denmark). Subsequently, immunoreactivity was detected with diaminobenzidine chromogen, and cell nuclei were counterstained with hematoxylin. The stained sections were digitalized using the Panoramic MIDI digital slide scanner at its highest resolution. Total images were captured with Pannoramic viewer software, and five microscopic focal fields (at × 200 magnification) were captured from each slide, and positive staining cells of the peritoneal abdomen layer (fibrosis occurred) were evaluated with *ImageJ* software with IHC toolbox plug-in (National Institutes of Health, Bethesda, MD, USA) [34].

### Immunofluorescent (IF) staining

Rat peritoneal tissue sections were prepared as aforementioned. In brief, after treated with primary antibodies (Table S1) for 2 h at room temperature, sections were washed and allowed to react with secondary antibody goat anti-mouse IgG conjugated with Alexa 546 or donkey anti-goat conjugated with Alexa 488 (Invitrogen, Carlsbad, CA) or goat anti-rabbit IgG conjugated with Cy3 (Sigma-Aldrich) for 45 min at room temperature. The slides were visualized with an anti-fading reagent containing DAPI (4′,6-diamidino-2-phenylindole) (Nakarai Tesque, Kyoto, Japan) for nuclear staining. They were digitalized using the Panoramic MIDI digital slide scanner at its highest resolution.

### Reagents

Recombinant human transforming growth factor-beta 1 (rhTGF-β1) (Cat No.: 240-B-002), recombinant human interleukin-6 (rhIL-6) (Cat No.: 206-IL-010), and recombinant human tumor necrosis factor-alpha (rhTNF-α) (Cat No.: 210-TA) were purchased from R&D Systems (R&D Systems, MN, USA). Rat monoclonal antibody (Cat No.: 501125) for neutralization of human IL-6 bioactivity was purchased from BioLegend (BioLegend, CA, USA).

### Collection of ADSC-CM and BM-MSC-CM

For the collection of conditioned media (CM), ADSC or BM-MSC were seeded in a 100 mm dish (Corning Life Sciences, MA, USA) at a density of 1 × 10^6^ cells in a culture medium. After 24 h of recombinant human TGF-β1 treatment, the supernatant was removed and replaced with a fresh culture medium. After another 24-h incubation, the CM was collected and was centrifuged for 10 min at 1500 g. The pellet containing cellular debris was discarded, and the supernatant (CM) sterile filtered (0.2 μm) before being stored at − 80 °C. For treating NR8383 cells with CM, the CM or control medium (IMDM) was mixed with the NR8383 culture medium (F12K), and the ratio of CM: F12K was 1: 2.

### Enzyme-linked immunosorbent assay (ELISA) assay of IL-6 concentration

The cytokine concentration within the CM was analyzed using a human IL-6 DuoSet ELISA kit (DY206, R&D Systems, MN, USA) with DuoSet Ancillary Reagent kit2 (DY008, R&D Systems, MN, USA) according to manufacturer’s instructions, and IL-6 concentration was normalized with the cell total protein. Cells were lysed with RIPA buffer, and the total protein was quantified with the BCA method.

### Quantitative real-time polymerase chain reaction

Cells were collected, and total RNA was isolated using TRIzol Reagent (Invitrogen, CA, USA). Up to 1.0 μg of total RNA was reversely transcribed to complementary DNA by MMLV high-performance reverse transcriptase according to the manufacturer’s instructions (Epicenter, UK). Quantitative real-time PCR (qPCR) was performed with Fast SYBR Green master mix (2×, Thermo Fisher Scientific, MA, USA) by QuantStudio 3 real-time PCR system (Applied Biosystems, MA, USA) to determine the relative gene expression profiles. The sequences of primers (Tri-I Biotech, Taiwan) for qPCR are listed in Table S2.

### Statistical analysis

All data for statistical analysis were presented as the mean ± standard error of the mean (SEM). One-way ANOVA and Fisher’s LSD multiple comparisons test were used to compare data from every two groups by the GraphPad Prism software (GraphPad Software, CA, USA). A *p* value of less than 0.05 was considered statistically significant.

## Results

### MGO-induced rat peritoneal fibrosis was attenuated by both ADSC and BM-MSC

**Fig. 2:**
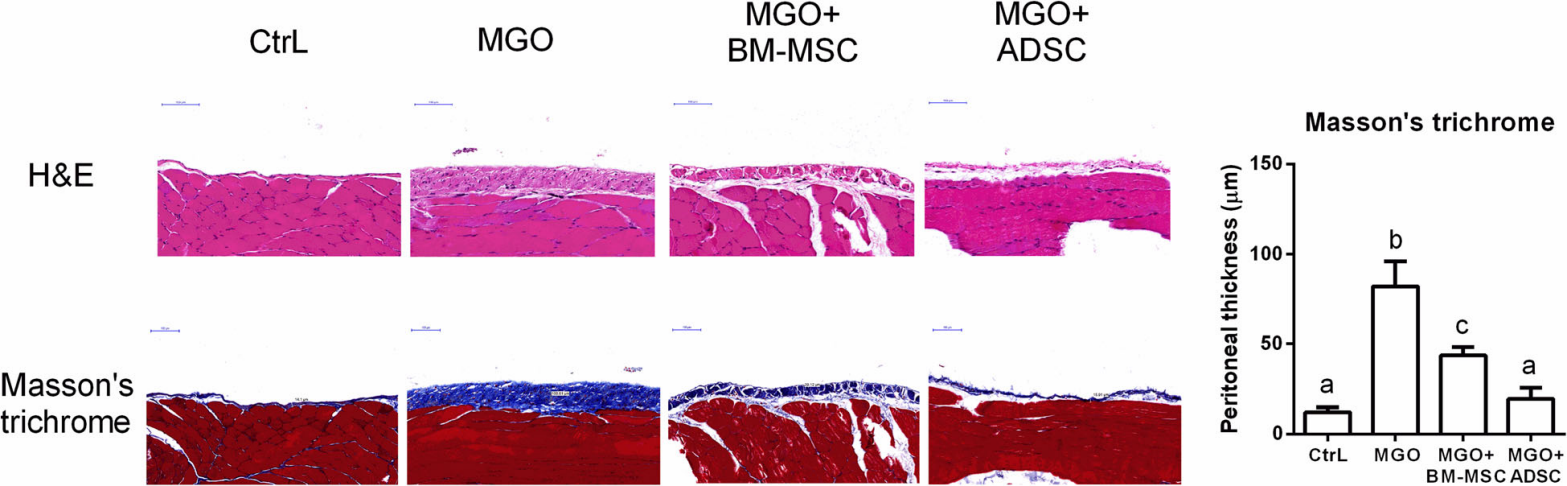
BM-MSC and ADSC reduced peritoneal fibrosis. Represented H&E stain and Masson’s trichrome stain photographs of four experimental groups include PBS controls (CtrL), peritoneal fibrosis induced by intraperitoneal injection of MGO, intraperitoneal MGO with intraperitoneal BM-MSC therapy (MGO+BM-MSC), and intraperitoneal MGO with intraperitoneal ADSC therapy (MGO+ADSC). Peritoneal thickness quantification was assessed from Masson’s trichrome stain. Data were presented as mean ± SEM. *n* = 6 for each groups. ANOVA, *p* < 0.05, different characters represent different levels of significance. Scale bar = 100 μm. Abbreviations: H&E hematoxylin and eosin, CtrL control, MGO methylglyoxal, BM-MSC bone marrow-derived mesenchymal stem cell, ADSC adipose-derived mesenchymal stem cell

**Fig. 3:**
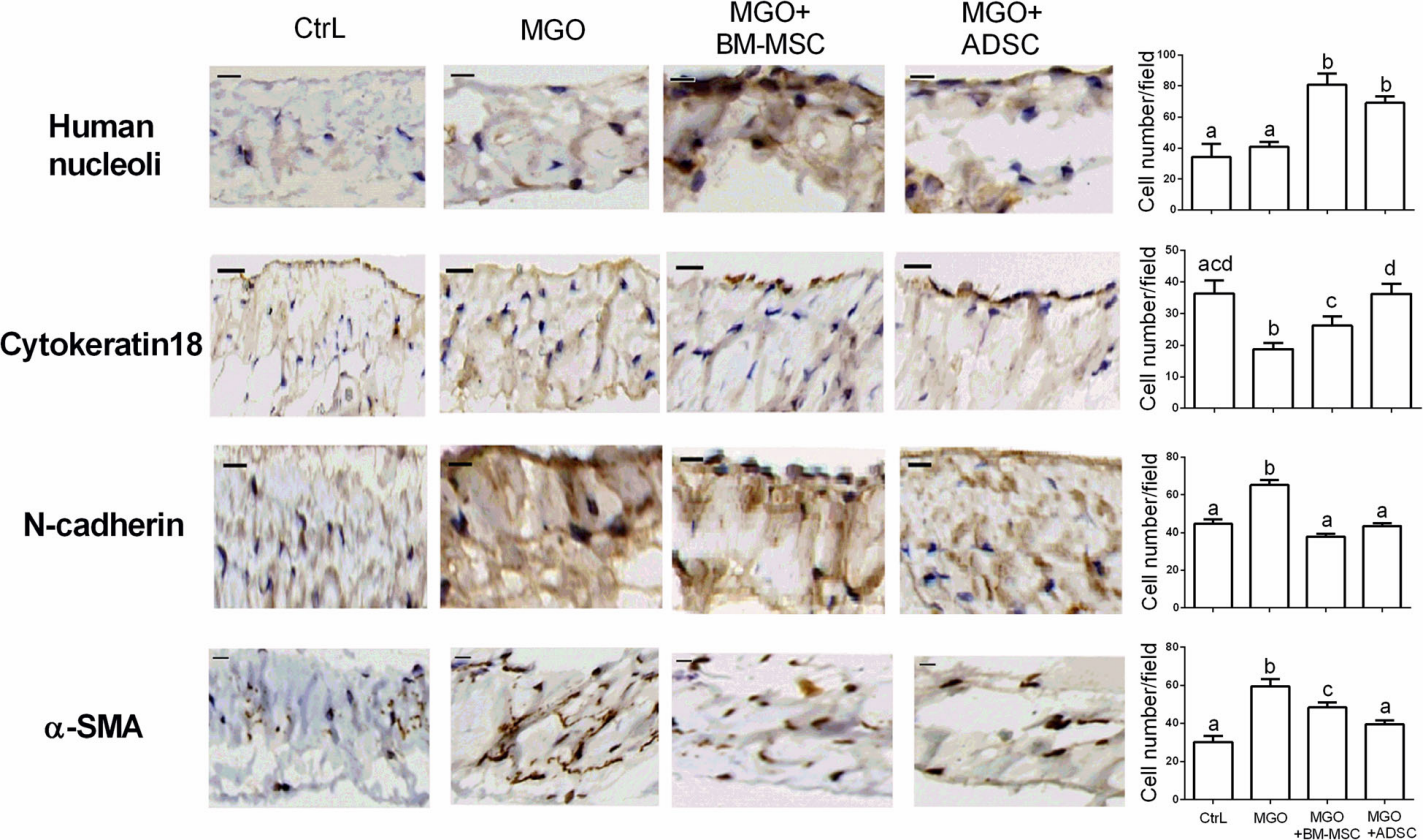
The dedifferentiation process of peritoneal mesothelial cells was reduced by intraperitoneal stem cell treatment. Represented IHC staining photographs were human nucleoli staining, to confirm the injection of human stem cells going to peritoneal abdomen layer; cytokeratin 18 staining, to indicate mesothelial cells of the peritoneal layer; N-cadherin staining, a mesenchymal cell marker of the peritoneal layer; α-SMA staining, to assess myofibroblast of the peritoneal layer. Cell nuclei were counterstained with hematoxylin. Data were presented as mean ± SEM. Control group *n* = 3 and the others *n* = 6. ANOVA, *p* < 0.05, different characters represent different levels of significance. Scale bar=50 μm. Abbreviations: CtrL control, MGO methylglyoxal, BM-MSC bone marrow-derived mesenchymal stem cell, ADSC adipose-derived mesenchymal stem cell, IHC immunohistochemistry, α-SMA alpha-smooth muscle actin

After 10 days of intraperitoneal injection of peritoneal dialysis buffer with MGO, we found that the rat peritoneum had a pallid appearance upon gross examination. The dialysis-related peritoneal thickening was induced by intraperitoneal MGO injection (Fig. 2, MGO vs. CtrL, *p* < 0.05). MGO-induced peritoneal thickening was attenuated by intraperitoneal injections of BM-MSC (Fig. 2, BM-MSC vs. MGO, *p* < 0.05) and ADSC (Fig. 2, ADSC vs. MGO, *p* < 0.05), respectively. Additionally, ADSC treatment showed a greater rescuing effect in terms of peritoneal thickness (Fig. 2, ADSC vs. CtrL, *p* > 0.05). The local injection of human stem cells, which appeared in the peritoneal mesothelial layer, was confirmed by human nucleoli IHC staining (Fig. 3).

### ADSC possessed more therapeutic effects than BM-MSC

In our animal experiment, ADSC showed more effectiveness than BM-MSC in attenuating dialysis-induced peritoneal thickening (Fig. 2, BM-MSC vs. ADSC, *p* < 0.05). The antifibrotic effect of ADSC was also more prominent than that of BM-MSC. The antifibrotic effect was assessed by Masson trichrome staining, IHC staining of mesothelial marker cytokeratin 18 (Fig. 3, BM-MSC vs. ADSC, *p* < 0.05) and myofibroblast marker α-SMA (Fig. 3, BM-MSC vs. ADSC, *p* < 0.05). As for N-cadherin, a mesenchymal cell marker, ADSC and BM-MSC showed a similar therapeutic effect (Fig. 3). The IF stains were consistent with the IHC results (Fig. S1).

### The therapeutic effect of ADSC was associated with peritoneal M2 macrophage polarization

**Fig. 4:**
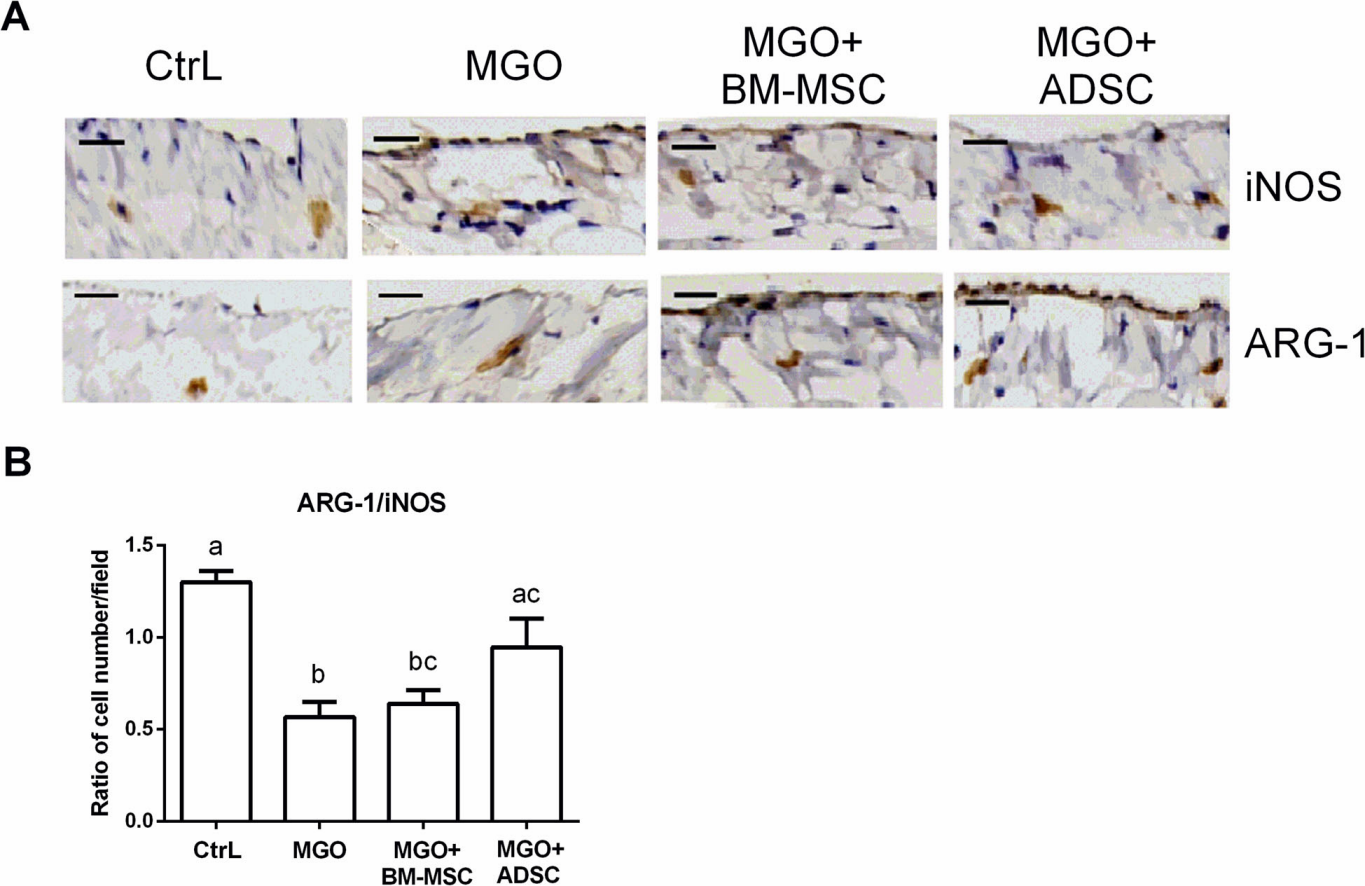
ADSC reversed macrophages M2/M1 ratio decreasing. **a** Represented IHC photographs were shown iNOS (M1 macrophage marker) staining and Arg-1 (M2 macrophage marker) staining. Cell nuclei were counterstained with hematoxylin. **b** The iNOS and Arg-1 stained cells were counted from IHC staining. Data were presented as mean ± SEM. Control group *n* = 3 and the others *n* = 6. ANOVA, *p* < 0.05, different characters represent different levels of significance. Scale bar = 50 μm. Abbreviations: CtrL control, MGO methylglyoxal, BM-MSC bone marrow-derived mesenchymal stem cell, ADSC adipose-derived mesenchymal stem cell, iNOS inducible nitric oxide synthase, Arg-1 arginase 1

**Fig. 5:**
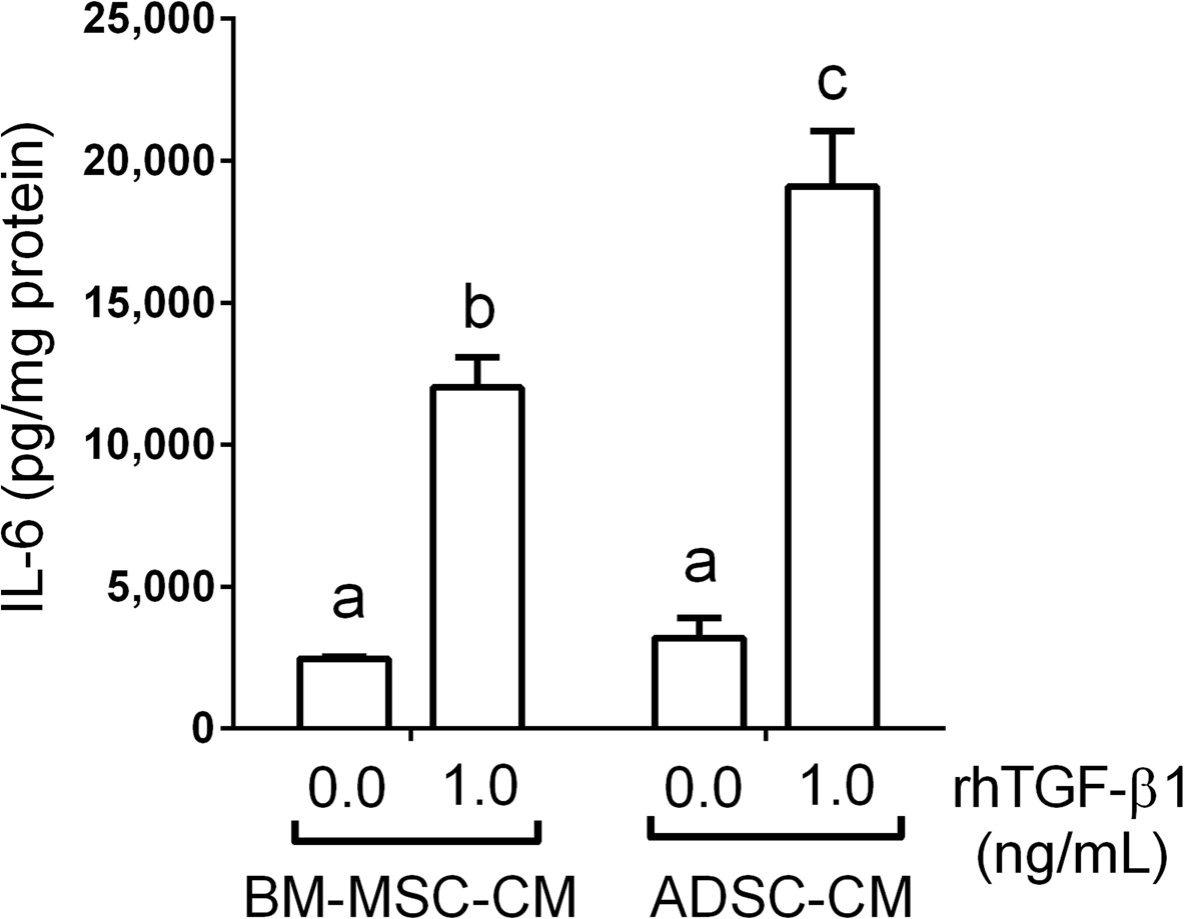
ADSC secreted more IL-6 upon TGF-β1 treatment. TGF-β1 treated with ADSC and BM-MSC for 24 h, then replaced with fresh medium without TGF-β1 and incubated for an additional 24 h. The conditioned medium (CM) of BM-MSC and ADSC was collected and analyzed with IL-6 ELISA. Data were presented as mean ± SEM. ANOVA, *p* < 0.05, different characters represent different levels of significance. Abbreviations: BM-MSC bone marrow-derived mesenchymal stem cell, ADSC adipose-derived mesenchymal stem cell, IL-6 interleukin-6, rhTGF-β1 recombinant human transforming growth factor-beta 1

The ratio of M2/M1 macrophage polarization (ARG-1/iNOS represented M2/M1 phenotype) in the peritoneal membrane was reduced in the MGO group, therefore peritoneal tissue M1 macrophage polarization (Fig. 4, MGO vs. CtrL, *p* < 0.05). ADSC treatment significantly reversed this process (Fig. 4, ADSC vs. MGO, *p* < 0.05; ADSC vs. CtrL, *p* > 0.05). BM-MSC treatment also showed a trend in reversing the peritoneal tissue ARG-1/iNOS ratio, but the difference did not achieve statistical significance (Fig. 5, BM-MSC vs. MGO, *p* > 0.05). The IF stains were consistent with the IHC results (Fig. S2).

### ADSC-CM upregulated more Arg-1 gene expression of macrophages than BM-MSC-CM did

**Fig. 6:**
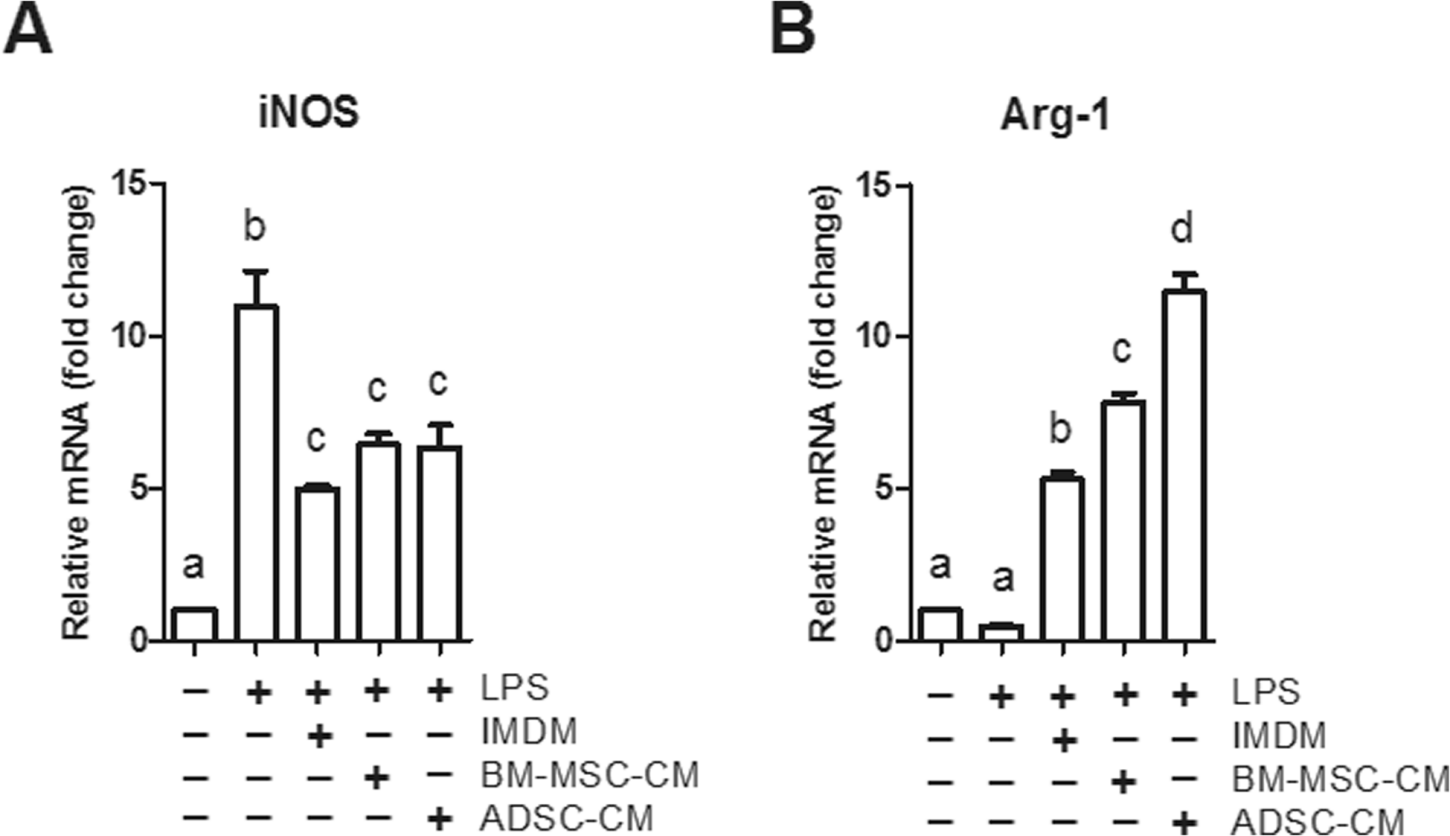
The conditioned medium (CM) of ADSC and BM-MSC induced Arg-1 gene expression of macrophage. NR8383 macrophages were treated with LPS (10.0 ng/mL) for 24 h and were then cultured with IMDM, ADSC-CM, or BM-MSC-CM for another 2 days. The cells iNOS (**a**) and Arg-1 (**b**) mRNA were analyzed by qPCR. Data were presented as mean ± SEM. ANOVA, *p* < 0.05, different characters represent different levels of significance. Abbreviations: BM-MSC bone marrow-derived mesenchymal stem cell, ADSC adipose-derived mesenchymal stem cell, iNOS inducible nitric oxide synthase, Arg-1 arginase 1, TGF-β1 transforming growth factor-beta 1, LPS lipopolysaccharides

According to our in vivo results, we hypothesized that ADSC-conditioned medium (ADSC-CM) upregulates Arg-1 and/or downregulates iNOS gene expression of macrophages to a greater extent compared to BM-MSC-CM. Therefore, we cultured NR8383 rat macrophage cells in the mixed medium, consisting of 2/3 F12K medium and 1/3 ADSC-CM or BM-MSC-CM, following determined the macrophage gene expression. In our in vitro experiments, we used lipopolysaccharides (LPS) to induce M1 macrophage polarization (Fig. 6A, LPS vs. no treatment, *p* < 0.05), which resembles MGO induced Arg-1/iNOS ratio reduction in the rat peritoneal tissue IHC images. The addition of IMDM, BM-MSC-CM, or ADSC-CM after LPS induction all downregulated iNOS expression (Fig. 6a, TGF-β1 vs. LPS, *p* < 0.05) and upregulated Arg-1 expression (Fig. 6b, IMDM vs. LPS, *p* < 0.05). BM-MSC-CM induced more Arg-1 expression than IMDM (Fig. 6b, BM-MSC-CM v.s IMDM, *p* < 0.05), and ADSC-CM induced even more Arg-1 expression than BM-MSC-CM (Fig. 6b, ADSC vs. BM-MSC-CM, *p* < 0.05). Intriguingly, BM-MSC-CM and ADSC-CM did not further downregulate iNOS expression as compared to IMDM (Fig. 6a).

### ADSC secreted more IL-6 upon TGF-β1 treatment

It has been reported that IL-6 secreted by MSC is capable of modulating macrophage polarization [35–39]. To address the superior effect of ADSC on peritoneal tissue macrophage polarization, a possible explanation is the higher level of IL-6 secreted by ADSC compared to BM-MSC under the same peritoneal inflammation environment. We treated both ADSC and BM-MSC with TGF-β1, a pleiotropic cytokine released in the peritoneal cavity and the main mediator of peritoneal fibrosis [40, 41], then measured IL-6 in the medium. These two stem cells were stimulated with 1.0 ng/mL TGF-β1 for 24 h, then cultured for another 24 h under the refreshed medium without TGF-β1. Lastly, and the supernatant was collected as the stem cell-CM. As shown in Fig. 5, both BM-MSC and ADSC secreted IL-6 upon TGF-β1 treatment (1.0 ng/mL vs. 0.0 ng/mL, *p* < 0.05). Furthermore, ADSC secreted more IL-6 than BM-MSC did after TGF-β1 treatment (ADSC vs. BM-MSC, *p* < 0.05). Fig. S3 shows that ADSC secreted more IL-6 than BM-MSC did (ADSC vs. BM-MSC, *p* < 0.05) upon various concentrations of TGF-β1 exposure (0–10 ng/mL). Next, IL-6 was then examined at the tissue level, and we found that rat peritoneal tissue IL-6 seems to be slightly higher in stem cell treatment groups. On the other hand, IF stains showed that tissue TGF-β1 was upregulated in the MGO-treated group as compared to the control. Both MSC-treated groups showed an attenuated TGF-β1 level in the mesothelial cell layer (Fig. S4).

### IL-6, as a key component of stem cell-conditioned media, promoted M2 macrophage polarization

**Fig. 7:**
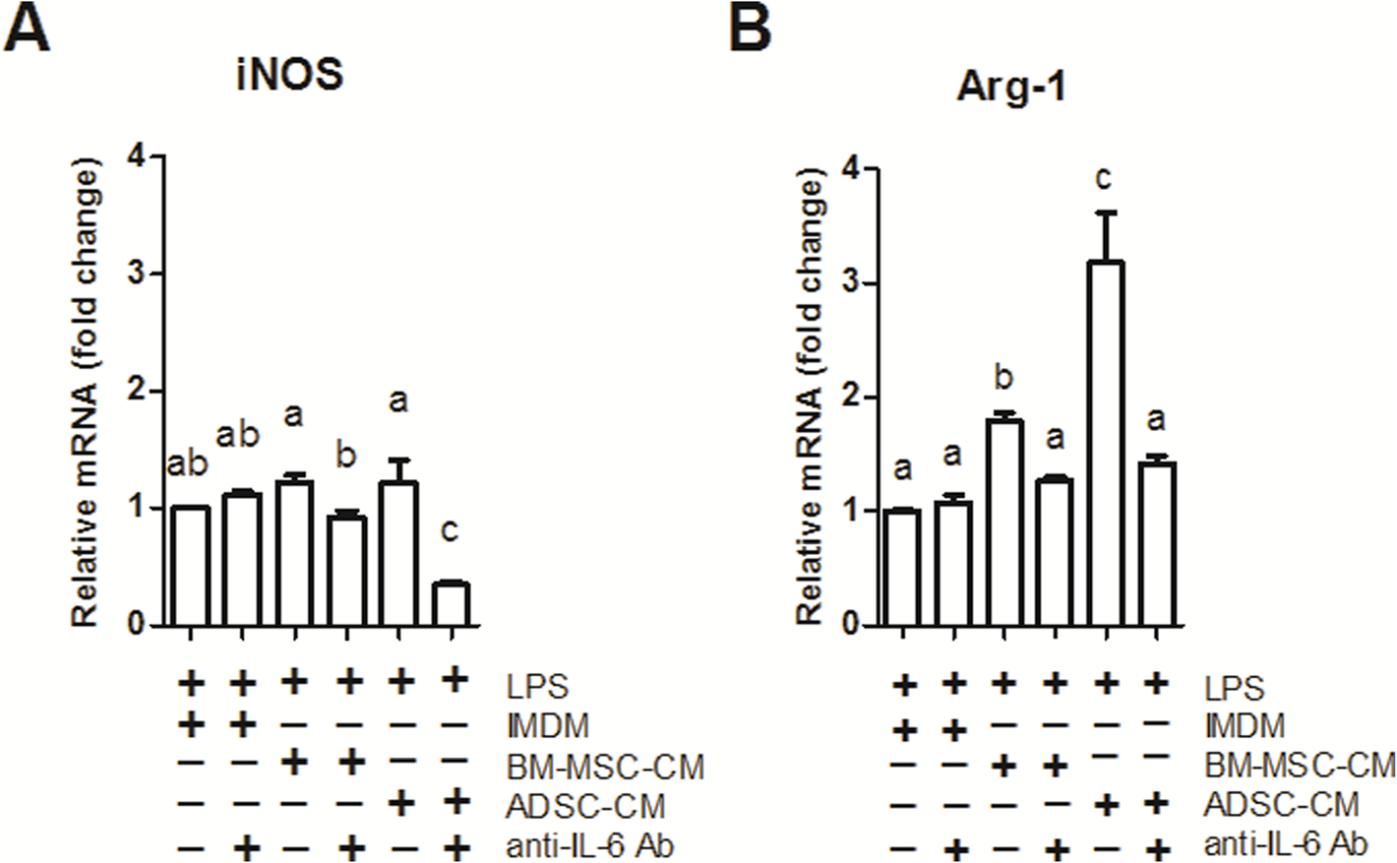
IL-6 neutralization downregulated macrophage iNOS and Arg-1 gene expression. NR8383 macrophages were treated with LPS plus ADSC-CM or BM-MSC-CM with/without IL-6 neutralizing antibody (1.0 μg/mL) for 3 days. The cells were analyzed by qPCR for iNOS (**a**) and Arg-1 (**b**) mRNA. Data were presented as mean ± SEM. ANOVA, *p* < 0.05, different characters represent different levels of significance. Abbreviations: BM-MSC bone marrow-derived mesenchymal stem cell, ADSC adipose-derived mesenchymal stem cell, iNOS inducible nitric oxide synthase, Arg-1 arginase-1, TGF-β1 transforming growth factor-beta 1, LPS lipopolysaccharides, IL-6 Ab interleukin-6 neutralizing antibody

As mentioned above, ADSC-CM secreted more IL-6 and was more competent to up-regulate macrophage Arg-1 expression as compared to BM-MSC-CM. We theorized that IL-6 is a key component of the MSC secretome that alters the behavior of macrophages. We neutralized IL-6 in stem cell-CM, and Arg-1 expression was downregulated to the greatest extent in ADSC-CM (Fig. 7b, BM-MSC-CM vs. IMDM, *p* < 0.05; ADSC-CM vs. IMDM, *p* < 0.05). Besides, ADSC-CM plus rhIL-6 upregulated Arg-1 expression to a greater extent than BM-MSC-CM plus rhIL-6 did (Fig. S5B, ADSC-CM vs. BM-MSC-CM, *p* < 0.05; BM-MSC-CM vs. CtrL, *p* < 0.05) in a dose-dependent manner (Fig. S5D). We also examined the control medium, IMDM. Neutralizing IL-6 in IMDM showed no effect on these two macrophage marker genes (Fig. S6, anti-IL-6 Ab vs. CtrL, *p* > 0.05). Next, we added different doses of TNF-α in ADSC-CM, which showed a trend to induce macrophage M1 polarization but not M2 (Fig. S7).

## Discussion

In this study, dialysis-related peritoneal thickening induced by intraperitoneal MGO injection could be rescued by intraperitoneal injection of either BM-MSC or ADSC. The antifibrotic effect of ADSC was significantly more prominent than that of BM-MSC. Previous studies have shown an interaction between ADSC and its niche signaling [42, 43]. Since peritoneal mesothelial cells are embedded in the adipose tissue of the abdominal cavity, the adipose-derived cells may be the more physiological and rational choice to provide cell signaling for peritoneal mesothelial cells and their surrounding immune cells.

TGF-β1, as a key fibrogenic factor, is induced in peritoneal mesothelial cells by exposing them to PD dialysate containing high concentrations of glucose [44]. To elucidate the molecular mechanism of our findings, we conducted in vitro experiments to investigate the beneficial component of the stem cell secretome. We found that upon TGF-β1 treatment, ADSC secreted more IL-6 than BM-MSC did. Furthermore, we found that IL-6, as a critical component of stem cell-CM, promotes M2 macrophage polarization. This observation is partly supported by previous studies focusing on tissue regeneration. In the mouse model of hindlimb ischemia and myocardial infarction, human MSC activates M2 macrophages through IL-6 secretion [38, 39]. Such IL-6-dependent M2 macrophage polarization is also seen in MSC-treated skin wound and ischemia-reperfusion kidney models [37, 45]. When placed in an inflammatory microenvironment, MSC immunomodulatory effect is enhanced partially through secretory factors [46–48]. Our dialysis-induced peritoneal inflammation model exerted immunomodulatory effects via IL-6 secretion after MSCs were injected into the peritoneal cavity. A study had demonstrated that, in contrast to the systemic effect of IL-6, local administration of IL-6 exerts an anti-inflammatory effect, provide evidence of the binary effect of IL-6 [49]. Besides, previous literature showed that, upon IL-6 exposure, the expression and response of IL-4 receptors in macrophages were upregulated, leading to STAT6 phosphorylation in a cell-autonomous manner [35, 50]. Macrophages were then polarized into the M2 phenotype and exerted anti-inflammatory properties [35, 50]. This article, to the best of our knowledge, is the first to reveal that under an inflammatory environment of TGF-β1, MSCs secrete more IL-6 to modulate peritoneal tissue M2 macrophage polarization.

We believe that the prominent M2 polarization effect of ADSC is the result of more IL-6 secretion. This inference was proved by M1 and M2 macrophage marker gene expression difference upon two stem cell-CM treatment (Fig. 6) as well as IL-6 neutralization (Fig. 7). Figs. 5 and 6 together show that amount of IL-6 in ADSC-CM is higher than that in BM-MSC-CM when accounting for the superior M2 polarizing ability of ADSC-CM. Meanwhile, IL-6 neutralizing experiments further confirmed that IL-6 was responsible for the ADSC-CM and MSC-CM-induced M2 polarization (Fig. 7b). As for the reduced iNOS expression upon IL-6 neutralization (Fig. 7a), it might reflect that macrophages require a basal IL-6 level to maintain iNOS expression.

Although IL-6 addition induced both iNOS and Arg-1 expression in control medium IMDM (Figs. S2 and S3), the iNOS expression did not increase in both stem cell-CMs as compared to IMDM alone (Fig. S2A). The addition of IL-6 in ADSC-CM even suppressed iNOS when compared with BM-MSC-CM and IMDM. This raised an interesting question about whether a higher dose of IL-6 in the CMs suppressed iNOS expression. Previous studies indicate that chronic low dose IL-6 tends to pro-inflammatory, whereas short-term/pulsatile high dose IL-6 tends to be anti-inflammatory [49, 51], which might explain our results.

The pathogenesis of dialysis-induced PF is a complex process. A combination of dedifferentiation of peritoneal mesothelial cells and chronic inflammation initiate and advance PF formation [8, 52–54]. Inflammatory macrophage (M1) populations participate in fibrosis, which suggests a requirement for inflammation in fibrosis development [55–58]. Our data showed a reduced M2/M1 ratio in MGO-induced peritoneal inflammation, consistent with more M1-like macrophage accumulation and/or M2-like macrophage reduction. Moreover, our data showed MSCs alleviated MGO-induced dedifferentiation of mesothelial cells to maintain epithelial integrity. ADSC and BM-MSC-induced functional improvement of injured tissues is mostly related to a paracrine effect rather than direct engraftment and differentiation [23, 59]. Besides, Shi et al. demonstrated that M1 macrophages induced dedifferentiation of peritoneal mesothelial cells in the in vitro co-culture experiment [60]. The present study further provides in vivo evidence showing that M2 macrophage polarization is associated with the inhibition of mesothelial cell dedifferentiation, supporting the antifibrotic effects of MSCs.

Macrophage phenotype switch has been observed in the past. MSCs cultured under both normoxic and hypoxic conditions release extracellular vesicles (EVs) endowed with anti-inflammatory effects [61]. When co-cultured with responding bone marrow-derived macrophages, both types of EVs were efficiently internalized by responding to bone marrow-derived macrophages, eliciting their switch from an M1 to an M2 phenotype [61]. Similarly, our data showed that ADSC reversed the lower M2/M1 ratio induced by MGO, and the effect of ADSC protected peritoneum from MGO damage. Whether this is caused by cytokines other than IL-6 and/or EVs is unclear and may require more studies. This effect was not seen on the BM-MSC we used.

In conclusion, our findings reveal a previously unidentified role of tissue macrophage in this antifibrotic process. ADSCs boost the M2-polarizing IL-6 secretion when situated in an inflammatory environment of TGF-β1, explaining the therapeutic mechanism of dialysis-induced peritoneal fibrosis. For a catastrophic and medical intractable illness such as encapsulating peritoneal sclerosis, local administration of autologous ADSC may be an alternative therapeutic path. Further studies are warranted, but the application values of ADSC are extremely promising.

## Supplementary Information

**Additional file 1: Figure S1.** The dedifferentiation process of peritoneal mesothelial cells was shown as represented immunofluorescence staining: human nucleoli, cytokeratin 18, N-cadherin staining, and α-SMA staining (red color). Cell nuclei were counterstained with DAPI (blue color). Scale bar = 50 μm. Abbreviations: CtrL, control; MGO, methylglyoxal; BM-MSC, bone marrow-derived mesenchymal stem cell; ADSC, adipose-derived mesenchymal stem cell; α-SMA, alpha-smooth muscle actin; DAPI, 4',6-diamidino-2-phenylindole. **Figure S2.** Macrophage polarization was shown as represented immunofluorescence staining: iNOS (M1 macrophage marker), Arg-1and CD163 (M2 macrophage markers) staining (red color). Cell nuclei were counterstained with DAPI (blue color). Scale bar = 50 μm. Abbreviations: CtrL, control; MGO, methylglyoxal; BM-MSC, bone marrow-derived mesenchymal stem cell; ADSC, adipose-derived mesenchymal stem cell; iNOS, inducible nitric oxide synthase; Arg-1, arginase 1; DAPI, 4',6-diamidino-2-phenylindole. **Figure S3.** ADSC secreted more IL-6 by TGF-β1 treatment. Different concentration of TGF-β1 was treated with BM-MSC and ADSC for 24 h, and the supernatant medium was analyzed with IL-6 ELISA. Data were presented as mean ± SEM. ANOVA, *p* < 0.05, different characters represent different levels of significance. Abbreviations: BM-MSC, bone marrow-derived mesenchymal stem cell; ADSC, adipose-derived mesenchymal stem cell; IL-6, interleukin-6; rhTGF-β1, recombinant human transforming growth factor-beta 1. **Figure S4.** The cytokines of the peritoneal mesothelial cell layer were shown as represented immunofluorescence staining: IL-6 (green color) and TGF-β1 (red color). Cell nuclei were counterstained with DAPI (blue color). Scale bar = 50 μm. Abbreviations: CtrL, control; MGO, methylglyoxal; BM-MSC, bone marrow-derived mesenchymal stem cell; ADSC, adipose-derived mesenchymal stem cell; IL-6, interleukin-6; TGF-β1, transforming growth factor-beta 1; DAPI, 4′,6-diamidino-2-phenylindole. **Figure S5.** Dose-dependent effect of IL-6 in ADSC-CM induced M2 polarization. NR8383 macrophages were treated with LPS plus ADSC-CM or BM-MSC-CM with/without IL-6 for three days. The cells were analyzed by qPCR for iNOS (A and C) and Arg-1 (B and D) mRNA. Data were presented as mean ± SEM. ANOVA, *p* < 0.05, different characters represent different levels of significance. Abbreviations: BM-MSC, bone marrow-derived mesenchymal stem cell; ADSC, adipose-derived mesenchymal stem cell; iNOS, inducible nitric oxide synthase; Arg-1, arginase-1; TGF-β1, transforming growth factor-beta 1; LPS, lipopolysaccharides; rhIL-6, recombinant human interleukin-6. **Figure S6.** Additional IL-6 induced iNOS and Arg-1 gene expression. NR8383 macrophages were treated with LPS plus IMDM with/without IL-6 (1.0 ng/mL) or IL-6 neutralizing antibody (1.0 μg/mL) for three days. The cells were analyzed by qPCR for iNOS (A) and Arg-1 (B) mRNA. Data were presented as mean ± SEM. ANOVA, *p* < 0.05, different characters represent different levels of significance. Abbreviations: iNOS, inducible nitric oxide synthase; Arg-1, arginase 1; LPS, lipopolysaccharides; IL-6, interleukin-6; anti-IL-6 Ab, IL-6 neutralizing antibody. **Figure S7.** Additional TNF-α did not up-regulate macrophage iNOS and Arg-1 gene expression. NR8383 macrophages were treated with LPS plus ADSC-CM with/without TNF-α for three days. The cells were analyzed by qPCR for iNOS (A) and Arg-1 (B) mRNA. Data were presented as mean ± SEM. ANOVA, *p* < 0.05, different characters represent different levels of significance. Abbreviations: ADSC, adipose-derived mesenchymal stem cell; iNOS, inducible nitric oxide synthase; Arg-1, arginase-1; LPS, lipopolysaccharides; rhTNF-α, recombinant human tumor necrosis factor-alpha. **Table S1.** Primary antibodies used for immunohistochemical and immunofluorescence staining. **Table S2.** Sequences of RT-PCR primers for macrophage marker genes.

## Data Availability

All data generated or analyzed during this study are included in this published article and its supplementary file.

## References

[1] Gotloib L, Shostak A, Wajsbrot V, Kushnier R (1999). High glucose induces a hypertrophic, senescent mesothelial cell phenotype after long in vivo exposure. Nephron.

[2] Yang AH, Chen JY, Lin YP, Huang TP, Wu CW (1997). Peritoneal dialysis solution induces apoptosis of mesothelial cells. Kidney Int.

[3] Jin HM, Di YD, Xu QJ (1999). Effects of commercial glucose-based peritoneal dialysates on peripheral blood phagocytes apoptosis. PeritDialInt.

[4] Aroeira LS, Aguilera A, Sanchez-Tomero JA, Bajo MA, del PG, Jimenez-Heffernan JA, Selgas R, Lopez-Cabrera M: Epithelial to mesenchymal transition and peritoneal membrane failure in peritoneal dialysis patients: pathologic significance and potential therapeutic interventions. JAmSocNephrol 2007, 18:2004–2013. 10.1681/ASN.2006111292 17568021

[5] Yung S, Chan TM (2012). Pathophysiological changes to the peritoneal membrane during PD-related peritonitis: the role of mesothelial cells. Mediat Inflamm.

[6] Devuyst O, Margetts PJ, Topley N (2010). The pathophysiology of the peritoneal membrane. J Am Soc Nephrol.

[7] Pletinck A, Vanholder R, Veys N, Van Biesen W (2012). Protecting the peritoneal membrane: factors beyond peritoneal dialysis solutions. Nat Rev Nephrol.

[8] Yang CY, Chau YP, Lee HT, Kuo HY, Lee OK, Yang AH (2013). Cannabinoid receptors as therapeutic targets for dialysis-induced peritoneal fibrosis. AmJNephrol.

[9] Murray LA, Rosada R, Moreira AP, Joshi A, Kramer MS, Hesson DP, Argentieri RL, Mathai S, Gulati M, Herzog EL, Hogaboam CM (2010). Serum amyloid P therapeutically attenuates murine bleomycin-induced pulmonary fibrosis via its effects on macrophages. PLoS One.

[10] Chu D, Du M, Hu X, Wu Q, Shen J (2011). Paeoniflorin attenuates schistosomiasis japonica-associated liver fibrosis through inhibiting alternative activation of macrophages. Parasitology.

[11] Murphy BS, Bush HM, Sundareshan V, Davis C, Hagadone J, Cory TJ, Hoy H, Hayes D, Anstead MI, Feola DJ (2010). Characterization of macrophage activation states in patients with cystic fibrosis. J Cyst Fibros.

[12] Cao Q, Wang Y, Harris DC (2014). Macrophage heterogeneity, phenotypes, and roles in renal fibrosis. Kidney Int Suppl (2011).

[13] Cao Q, Harris DC, Wang Y (2015). Macrophages in kidney injury, inflammation, and fibrosis. Physiology (Bethesda).

[14] Duffield JS (2010). Macrophages and immunologic inflammation of the kidney. Semin Nephrol.

[15] Wynn TA, Barron L (2010). Macrophages: master regulators of inflammation and fibrosis. Semin Liver Dis.

[16] Pittenger MF, Mackay AM, Beck SC, Jaiswal RK, Douglas R, Mosca JD, Moorman MA, Simonetti DW, Craig S, Marshak DR (1999). Multilineage potential of adult human mesenchymal stem cells. Science.

[17] Wei X, Yang X, Han ZP, Qu FF, Shao L, Shi YF (2013). Mesenchymal stem cells: a new trend for cell therapy. Acta Pharmacol Sin.

[18] Ma S, Xie N, Li W, Yuan B, Shi Y, Wang Y (2014). Immunobiology of mesenchymal stem cells. Cell Death Differ.

[19] Tsai YA, Liu RS, Lirng JF, Yang BH, Chang CH, Wang YC, Wu YS, Ho JH, Lee OK, Soong BW (2017). Treatment of spinocerebellar ataxia with mesenchymal stem cells: a phase I/IIa clinical study. Cell Transplant.

[20] Strioga M, Viswanathan S, Darinskas A, Slaby O, Michalek J (2012). Same or not the same? Comparison of adipose tissue-derived versus bone marrow-derived mesenchymal stem and stromal cells. Stem Cells Dev.

[21] Ivanova-Todorova E, Bochev I, Mourdjeva M, Dimitrov R, Bukarev D, Kyurkchiev S, Tivchev P, Altunkova I, Kyurkchiev DS (2009). Adipose tissue-derived mesenchymal stem cells are more potent suppressors of dendritic cells differentiation compared to bone marrow-derived mesenchymal stem cells. Immunol Lett.

[22] Noel D, Caton D, Roche S, Bony C, Lehmann S, Casteilla L, Jorgensen C, Cousin B (2008). Cell specific differences between human adipose-derived and mesenchymal-stromal cells despite similar differentiation potentials. Exp Cell Res.

[23] Wakabayashi K, Hamada C, Kanda R, Nakano T, Io H, Horikoshi S, Tomino Y (2014). Adipose-derived mesenchymal stem cells transplantation facilitate experimental peritoneal fibrosis repair by suppressing epithelial-mesenchymal transition. J Nephrol.

[24] Ueno T, Nakashima A, Doi S, Kawamoto T, Honda K, Yokoyama Y, Doi T, Higashi Y, Yorioka N, Kato Y, Kohno N, Masaki T (2013). Mesenchymal stem cells ameliorate experimental peritoneal fibrosis by suppressing inflammation and inhibiting TGF-beta1 signaling. Kidney Int.

[25] Tulpar S, Poyrazoglu MH, Ozbilge H, Bastug F, Gunduz Z, Torun YA, Kaya EG, Akgun H, Dursun I, Dusunsel R (2012). Modulation of inflammation by mesenchymal stem cell transplantation in peritoneal dialysis in rats. Ren Fail.

[26] Sekiguchi Y, Hamada C, Ro Y, Nakamoto H, Inaba M, Shimaoka T, Io H, Koyanagi I, Aruga S, Inuma J, Kaneko K, Hotta Y, Margetts PJ, Mochizuki H, Horikoshi S, Tomino Y (2012). Differentiation of bone marrow-derived cells into regenerated mesothelial cells in peritoneal remodeling using a peritoneal fibrosis mouse model. J Artif Organs.

[27] Costalonga EC, Fanelli C, Garnica MR, Noronha IL (2020). Adipose-derived mesenchymal stem cells modulate fibrosis and inflammation in the peritoneal fibrosis model developed in uremic rats. Stem Cells Int.

[28] Dykstra JA, Facile T, Patrick RJ, Francis KR, Milanovich S, Weimer JM, Kota DJ (2017). Concise review: fat and furious: harnessing the full potential of adipose-derived stromal vascular fraction. Stem Cells Transl Med.

[29] Lee CW, Hsiao WT, Lee OK (2017). Mesenchymal stromal cell-based therapies reduce obesity and metabolic syndromes induced by a high-fat diet. Transl Res.

[30] Chiang YH, Lin CC, Chen YC, Lee OK. Treatment of arsenite intoxication-induced peripheral vasculopathy with mesenchymal stem cells. Int J Mol Sci. 2018;19(4). 10.3390/ijms19041026. 10.3390/ijms19041026 PMC5979449 29596344

[31] Tsao YT, Shih YY, Liu YA, Liu YS, Lee OK (2017). Knockdown of SLC41A1 magnesium transporter promotes mineralization and attenuates magnesium inhibition during osteogenesis of mesenchymal stromal cells. Stem Cell Res Ther.

[32] Hirahara I, Ishibashi Y, Kaname S, Kusano E, Fujita T (2009). Methylglyoxal induces peritoneal thickening by mesenchymal-like mesothelial cells in rats. Nephrol Dial Transplant.

[33] Hirahara I, Kusano E, Yanagiba S, Miyata Y, Ando Y, Muto S, Asano Y (2006). Peritoneal injury by methylglyoxal in peritoneal dialysis. Perit Dial Int.

[34] Shu J, Dolman GE, Duan J, Qiu G, Ilyas M (2016). Statistical colour models: an automated digital image analysis method for quantification of histological biomarkers. Biomed Eng Online.

[35] Mauer J, Chaurasia B, Goldau J, Vogt MC, Ruud J, Nguyen KD, Theurich S, Hausen AC, Schmitz J, Bronneke HS (2014). Signaling by IL-6 promotes alternative activation of macrophages to limit endotoxemia and obesity-associated resistance to insulin. Nat Immunol.

[36] Ma Y, Gao M, Sun H, Liu D (1852). Interleukin-6 gene transfer reverses body weight gain and fatty liver in obese mice. Biochim Biophys Acta.

[37] Zhang QZ, Su WR, Shi SH, Wilder-Smith P, Xiang AP, Wong A, Nguyen AL, Kwon CW, Le AD (2010). Human gingiva-derived mesenchymal stem cells elicit polarization of m2 macrophages and enhance cutaneous wound healing. Stem Cells.

[38] Czapla J, Matuszczak S, Wisniewska E, Jarosz-Biej M, Smolarczyk R, Cichon T, Glowala-Kosinska M, Sliwka J, Garbacz M, Szczypior M (2016). Human cardiac nesenchymal stromal cells with CD105+CD34- phenotype enhance the function of post-infarction heart in mice. PLoS One.

[39] Pilny E, Smolarczyk R, Jarosz-Biej M, Hadyk A, Skorupa A, Ciszek M, Krakowczyk L, Kulach N, Gillner D, Sokol M (2019). Human ADSC xenograft through IL-6 secretion activates M2 macrophages responsible for the repair of damaged muscle tissue. Stem Cell Res Ther.

[40] Zhou Q, Bajo MA, Del Peso G, Yu X, Selgas R (2016). Preventing peritoneal membrane fibrosis in peritoneal dialysis patients. Kidney Int.

[41] Strippoli R, Moreno-Vicente R, Battistelli C, Cicchini C, Noce V, Amicone L, Marchetti A, Del Pozo MA, Tripodi M (2016). Molecular mechanisms underlying peritoneal EMT and fibrosis. Stem Cells Int.

[42] Patel RS, Carter G, El Bassit G, Patel AA, Cooper DR, Murr M, Patel NA (2016). Adipose-derived stem cells from lean and obese humans show depot specific differences in their stem cell markers, exosome contents and senescence: role of protein kinase C delta (PKCdelta) in adipose stem cell niche. Stem Cell Investig.

[43] Carter G, Apostolatos A, Patel R, Mathur A, Cooper D, Murr M, Patel NA (2013). Dysregulated alternative splicing pattern of PKCdelta during differentiation of human preadipocytes represents distinct differences between lean and obese adipocytes. ISRN Obes.

[44] Kang DH, Hong YS, Lim HJ, Choi JH, Han DS, Yoon KI (1999). High glucose solution and spent dialysate stimulate the synthesis of transforming growth factor-beta1 of human peritoneal mesothelial cells: effect of cytokine costimulation. PeritDialInt.

[45] Wise AF, Williams TM, Kiewiet MB, Payne NL, Siatskas C, Samuel CS, Ricardo SD (2014). Human mesenchymal stem cells alter macrophage phenotype and promote regeneration via homing to the kidney following ischemia-reperfusion injury. Am J Physiol Renal Physiol.

[46] Ren G, Zhao X, Wang Y, Zhang X, Chen X, Xu C, Yuan ZR, Roberts AI, Zhang L, Zheng B, Wen T, Han Y, Rabson AB, Tischfield JA, Shao C, Shi Y (2012). CCR2-dependent recruitment of macrophages by tumor-educated mesenchymal stromal cells promotes tumor development and is mimicked by TNFalpha. Cell Stem Cell.

[47] Rodriguez TM, Saldias A, Irigo M, Zamora JV, Perone MJ, Dewey RA (2015). Effect of TGF-beta1 stimulation on the secretome of human adipose-derived mesenchymal stromal cells. Stem Cells Transl Med.

[48] Philipp D, Suhr L, Wahlers T, Choi YH, Paunel-Gorgulu A (2018). Preconditioning of bone marrow-derived mesenchymal stem cells highly strengthens their potential to promote IL-6-dependent M2b polarization. Stem Cell Res Ther.

[49] Bhargava R, Janssen W, Altmann C, Andres-Hernando A, Okamura K, Vandivier RW, Ahuja N, Faubel S (2013). Intratracheal IL-6 protects against lung inflammation in direct, but not indirect, causes of acute lung injury in mice. PLoS One.

[50] Xie Z, Hao H, Tong C, Cheng Y, Liu J, Pang Y, Si Y, Guo Y, Zang L, Mu Y, Han W (2016). Human umbilical cord-derived mesenchymal stem cells elicit macrophages into an anti-inflammatory phenotype to alleviate insulin resistance in type 2 diabetic rats. Stem Cells.

[51] Cox AA, Sagot Y, Hedou G, Grek C, Wilkes T, Vinik AI, Ghatnekar G (2017). Low-dose pulsatile interleukin-6 as a treatment option for diabetic peripheral neuropathy. Front Endocrinol (Lausanne).

[52] Chen T, Nie H, Gao X, Yang J, Pu J, Chen Z, Cui X, Wang Y, Wang H, Jia G (2014). Epithelial-mesenchymal transition involved in pulmonary fibrosis induced by multi-walled carbon nanotubes via TGF-beta/Smad signaling pathway. Toxicol Lett.

[53] Wermuth PJ, Jimenez SA (2015). The significance of macrophage polarization subtypes for animal models of tissue fibrosis and human fibrotic diseases. Clin Transl Med.

[54] Yang CY, Chau YP, Chen A, Lee OK, Tarng DC, Yang AH (2017). Targeting cannabinoid signaling for peritoneal dialysis-induced oxidative stress and fibrosis. World J Nephrol.

[55] Guilherme RF, Xisto DG, Kunkel SL, Freire-de-Lima CG, Rocco PR, Neves JS, Fierro IM, Canetti C, Benjamim CF (2013). Pulmonary antifibrotic mechanisms aspirin-triggered lipoxin A (4) synthetic analog. Am J Respir Cell Mol Biol.

[56] Misson P, van den Brule S, Barbarin V, Lison D, Huaux F (2004). Markers of macrophage differentiation in experimental silicosis. J Leukoc Biol.

[57] Wijesundera KK, Izawa T, Tennakoon AH, Murakami H, Golbar HM, Katou-Ichikawa C, Tanaka M, Kuwamura M, Yamate J (2014). M1- and M2-macrophage polarization in rat liver cirrhosis induced by thioacetamide (TAA), focusing on Iba1 and galectin-3. Exp Mol Pathol.

[58] Wijesundera KK, Izawa T, Murakami H, Tennakoon AH, Golbar HM, Kato-Ichikawa C, Tanaka M, Kuwamura M, Yamate J (2014). M1- and M2-macrophage polarization in thioacetamide (TAA)-induced rat liver lesions; a possible analysis for hepato-pathology. Histol Histopathol.

[59] Bandeira F, Goh TW, Setiawan M, Yam GH, Mehta JS (2020). Cellular therapy of corneal epithelial defect by adipose mesenchymal stem cell-derived epithelial progenitors. Stem Cell Res Ther.

[60] Shi J, Li Q, Sheng M, Zheng M, Yu M, Zhang L (2016). The role of TLR4 in M1 macrophage-induced epithelial-mesenchymal transition of peritoneal mesothelial cells. Cell Physiol Biochem.

[61] Lo Sicco C, Reverberi D, Balbi C, Ulivi V, Principi E, Pascucci L, Becherini P, Bosco MC, Varesio L, Franzin C, Pozzobon M, Cancedda R, Tasso R (2017). Mesenchymal stem cell-derived extracellular vesicles as mediators of anti-inflammatory effects: endorsement of macrophage polarization. Stem Cells Transl Med.

