# Supplementary material for: Adipose-derived mesenchymal stem cells attenuate dialysis-induced peritoneal fibrosis by modulating macrophage polarization via interleukin-6
Source: Stem Cell Res Ther. 2021 Mar 19;12:193. doi: 10.1186/s13287-021-02270-4 (PMC7977319; doi:10.1186/s13287-021-02270-4)
Supplement: Supplementary file 1 — Additional file 1: Figure S1. The dedifferentiation process of peritoneal mesothelial cells was shown as represented immunofluorescence staining: human nucleoli, cytokeratin 18, N-cadherin staining, and α-SMA staining (red color). Cell nuclei were counterstained with DAPI (blue color). Scale bar = 50 μm. Abbreviations: CtrL, control; MGO, methylglyoxal; BM-MSC, bone marrow-derived mesenchymal stem cell; ADSC, adipose-derived mesenchymal stem cell; α-SMA, alpha-smooth muscle actin; DAPI, 4',6-diamidino-2-phenylindole. Figure S2. Macrophage polarization was shown as represented immunofluorescence staining: iNOS (M1 macrophage marker), Arg-1and CD163 (M2 macrophage markers) staining (red color). Cell nuclei were counterstained with DAPI (blue color). Scale bar = 50 μm. Abbreviations: CtrL, control; MGO, methylglyoxal; BM-MSC, bone marrow-derived mesenchymal stem cell; ADSC, adipose-derived mesenchymal stem cell; iNOS, inducible nitric oxide synthase; Arg-1, arginase 1; DAPI, 4',6-diamidino-2-phenylindole. Figure S3. ADSC secreted more IL-6 by TGF-β1 treatment. Different concentration of TGF-β1 was treated with BM-MSC and ADSC for 24 h, and the supernatant medium was analyzed with IL-6 ELISA. Data were presented as mean ± SEM. ANOVA, p < 0.05, different characters represent different levels of significance. Abbreviations: BM-MSC, bone marrow-derived mesenchymal stem cell; ADSC, adipose-derived mesenchymal stem cell; IL-6, interleukin-6; rhTGF-β1, recombinant human transforming growth factor-beta 1. Figure S4. The cytokines of the peritoneal mesothelial cell layer were shown as represented immunofluorescence staining: IL-6 (green color) and TGF-β1 (red color). Cell nuclei were counterstained with DAPI (blue color). Scale bar = 50 μm. Abbreviations: CtrL, control; MGO, methylglyoxal; BM-MSC, bone marrow-derived mesenchymal stem cell; ADSC, adipose-derived mesenchymal stem cell; IL-6, interleukin-6; TGF-β1, transforming growth factor-beta 1; DAPI, 4′,6-diamidino-2-phenylin [file 13287_2021_2270_MOESM1_ESM.docx]

**SUPPLEMENTARY MATERIALS**

**Figure S1. The dedifferentiation process of peritoneal mesothelial cells was shown as represented immunofluorescence staining: human nucleoli, cytokeratin 18, N-cadherin staining, and α-SMA staining (red color). Cell nuclei were counterstained with** **DAPI (blue color). Scale bar = 50 μm. Abbreviations: CtrL, control; MGO, methylglyoxal; BM-MSC, bone marrow-derived mesenchymal stem cell; ADSC, adipose-derived mesenchymal stem cell; α-SMA, alpha-smooth muscle actin;** **DAPI****, 4',6-diamidino-2-phenylindole.**

**
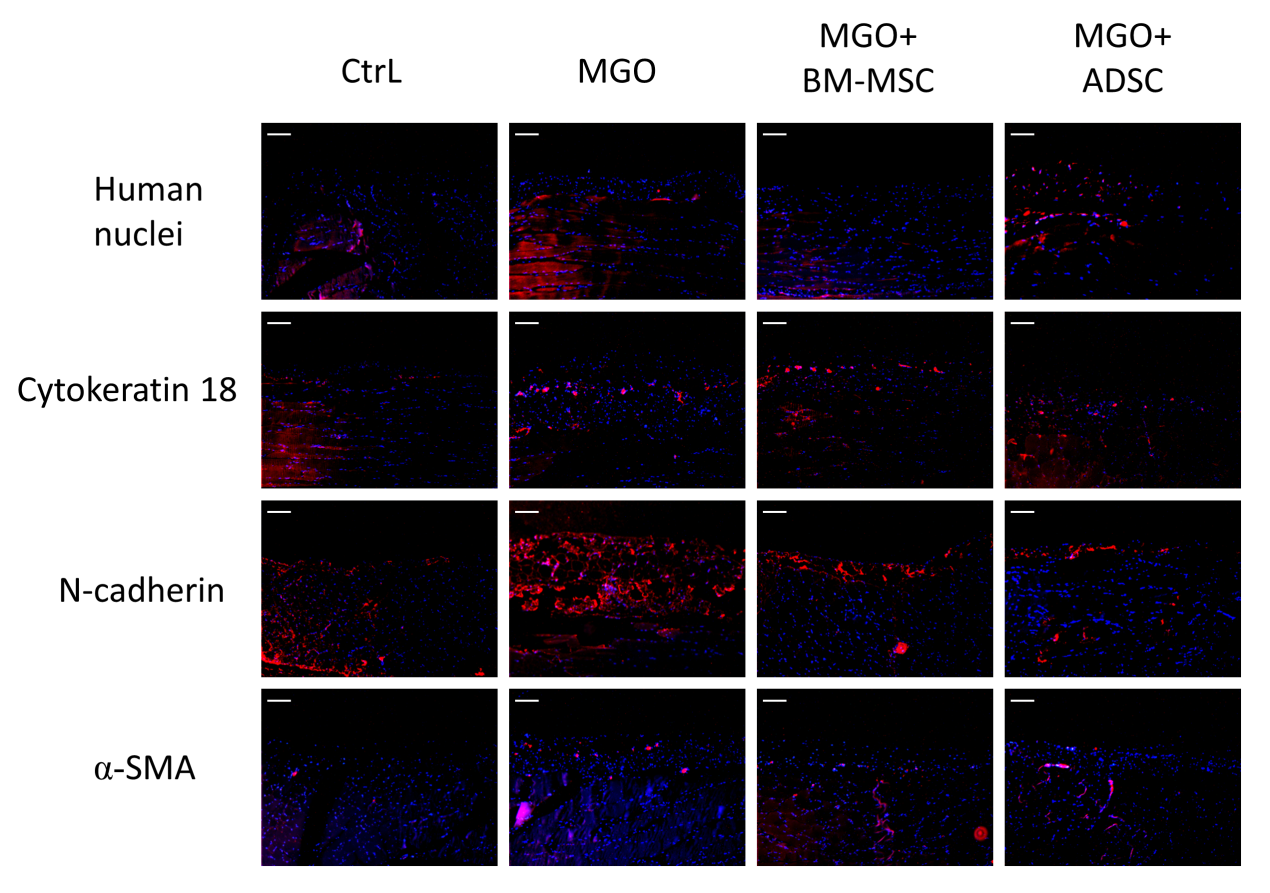
**

**Figure S2.** **Macrophage polarization was shown as represented immunofluorescence staining: iNOS (M1 macrophage marker), Arg-1and CD163 (M2 macrophage markers) staining** **(red color). Cell nuclei were counterstained with DAPI (blue color). Scale bar = 50 μm. Abbreviations: CtrL, control; MGO, methylglyoxal; BM-MSC, bone marrow-derived mesenchymal stem cell; ADSC, adipose-derived mesenchymal stem cell; iNOS, inducible nitric oxide synthase; Arg-1, arginase 1; DAPI, 4',6-diamidino-2-phenylindole.**

**
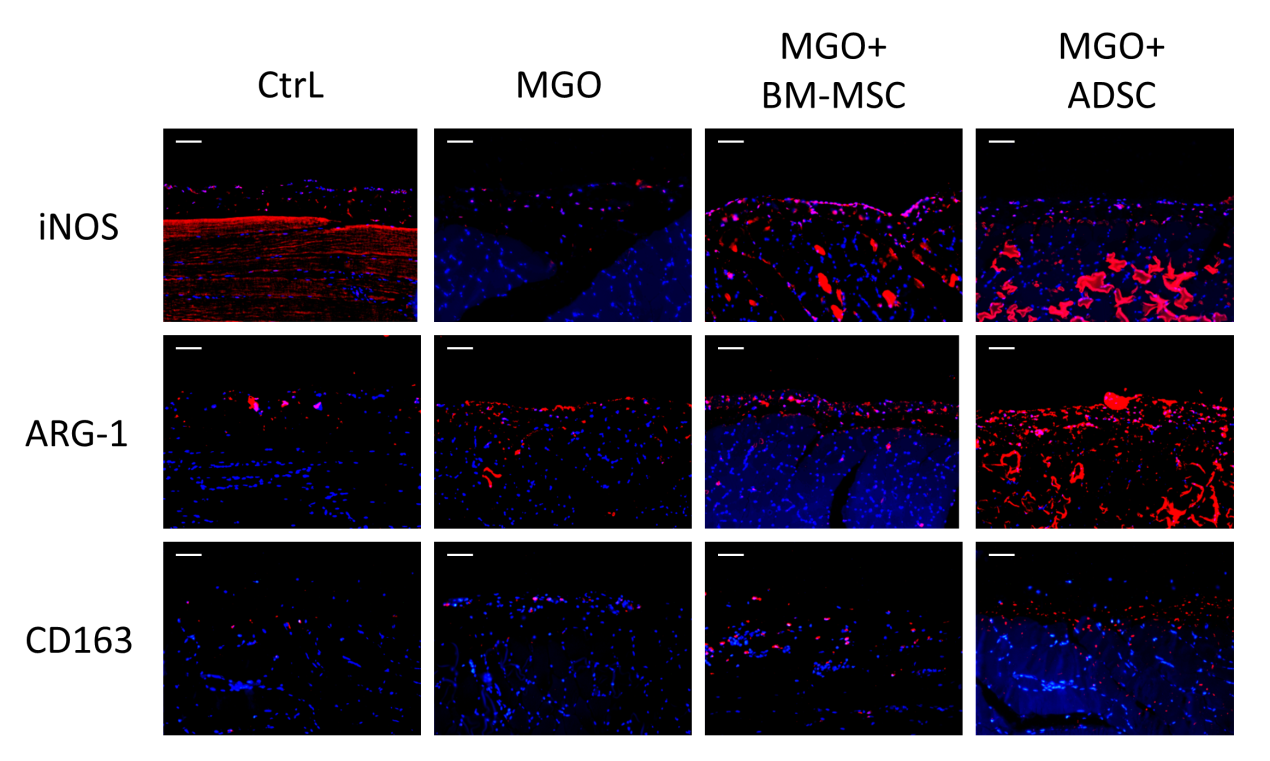
**

**Figure S3.** ADSC secreted more IL-6 by TGF-β1 treatment. Different concentration of TGF-β1 was treated with BM-MSC and ADSC for 24 hours, and the supernatant medium was analyzed with IL-6 ELISA. Data were presented as mean ± SEM. ANOVA, *p* < 0.05, different characters represent different levels of significance. Abbreviations: BM-MSC, bone marrow-derived mesenchymal stem cell; ADSC, adipose-derived mesenchymal stem cell; IL-6, interleukin-6; rhTGF-β1, recombinant human transforming growth factor-beta 1.


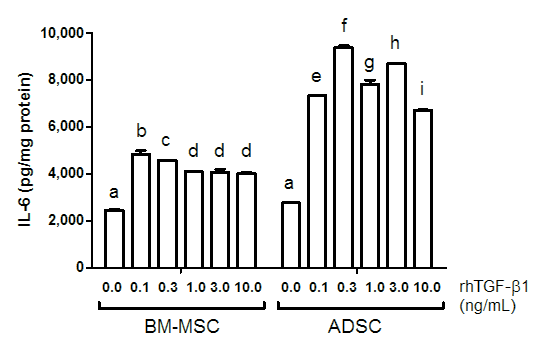


**Figure S4. The cytokines of the peritoneal mesothelial cell layer were shown as represented immunofluorescence staining: IL-6 (green color) and TGF-β1 (red color). Cell nuclei were counterstained with DAPI (blue color). Scale bar = 50 μm. Abbreviations: CtrL, control; MGO, methylglyoxal; BM-MSC, bone marrow-derived mesenchymal stem cell; ADSC, adipose-derived mesenchymal stem cell;** **IL-6, interleukin-6; TGF-β1, transforming growth factor-beta 1; DAPI, 4',6-diamidino-2-phenylindole.**

**
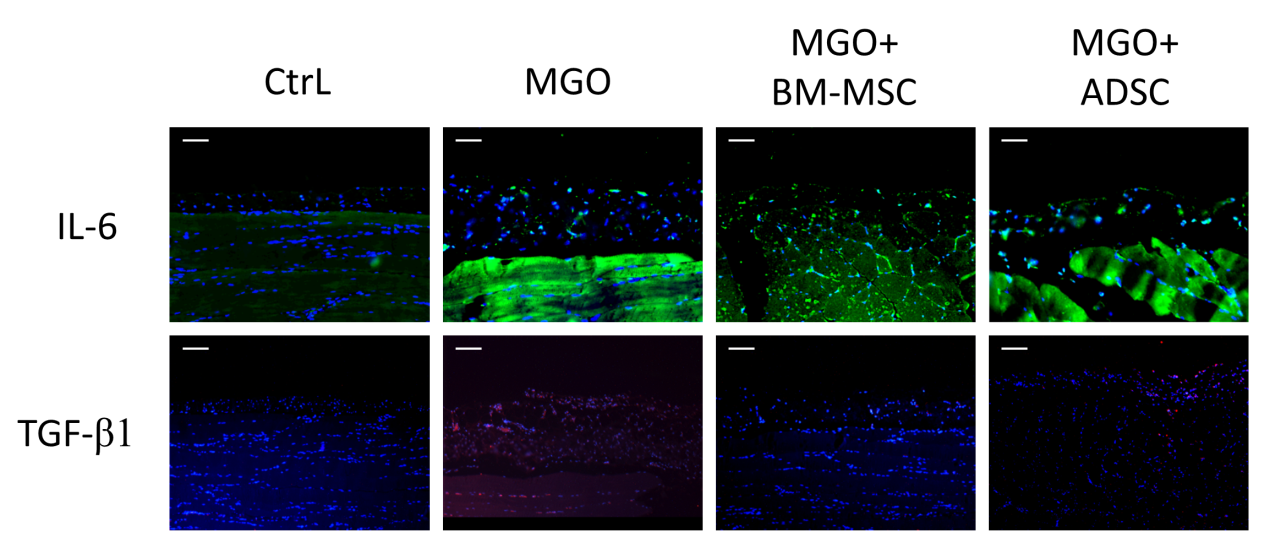
Figure S5.** Dose-dependent effect of IL-6 in ADSC-CM induced M2 polarization. NR8383 macrophages were treated with LPS plus ADSC-CM or BM-MSC-CM with/without IL-6 for three days. The cells were analyzed by qPCR for iNOS (A and C) and Arg-1 (B and D) mRNA. Data were presented as mean ± SEM. ANOVA, *p* < 0.05, different characters represent different levels of significance. Abbreviations: BM-MSC, bone marrow-derived mesenchymal stem cell; ADSC, adipose-derived mesenchymal stem cell; iNOS, inducible nitric oxide synthase; Arg-1, arginase-1; TGF-β1, transforming growth factor-beta 1; LPS, lipopolysaccharides; rhIL-6, recombinant human interleukin-6.


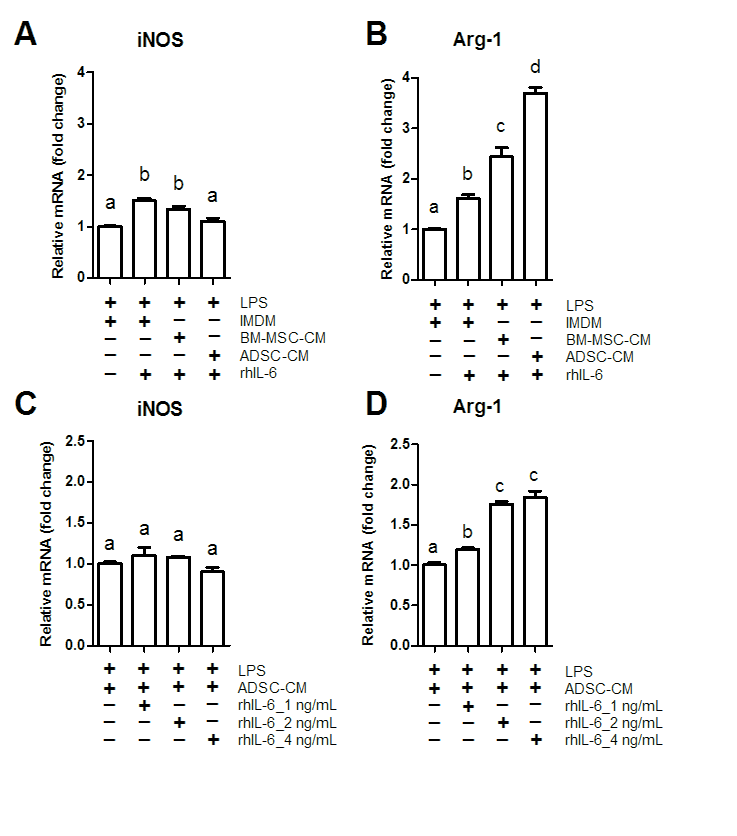


**Figure S6.** Additional IL-6 induced iNOS and Arg-1 gene expression. NR8383 macrophages were treated with LPS plus IMDM with/without IL-6 (1.0 ng/mL) or IL-6 neutralizing antibody (1.0 μg/mL) for three days. The cells were analyzed by qPCR for iNOS (A) and Arg-1 (B) mRNA. Data were presented as mean ± SEM. ANOVA, *p* < 0.05, different characters represent different levels of significance. Abbreviations: iNOS, inducible nitric oxide synthase; Arg-1, arginase 1; LPS, lipopolysaccharides; IL-6, interleukin-6; anti-IL-6 Ab, IL-6 neutralizing antibody.


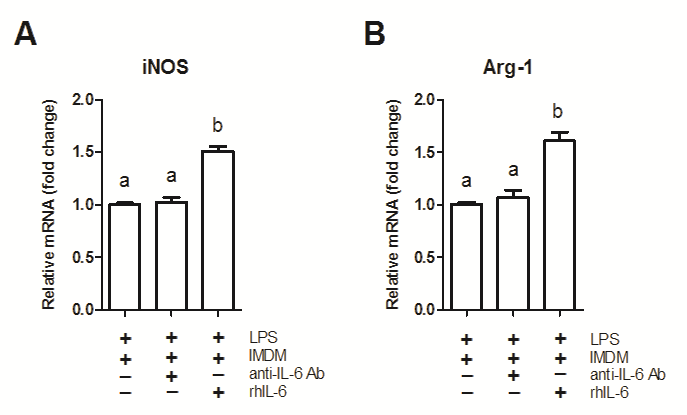


**Figure S7. Additional** **TNF-α did not up-regulate macrophage iNOS and Arg-1 gene expression. NR8383 macrophages were treated with LPS plus ADSC-CM with/without TNF-α for three days. The cells were analyzed by qPCR for iNOS (A) and Arg-1 (B) mRNA. Data were presented as mean ± SEM. ANOVA, *p* < 0.05, different characters represent different levels of significance. Abbreviations: ADSC, adipose-derived mesenchymal stem cell; iNOS, inducible nitric oxide synthase; Arg-1, arginase-1; LPS, lipopolysaccharides; rhTNF-α, recombinant human tumor necrosis factor-alpha.**

**
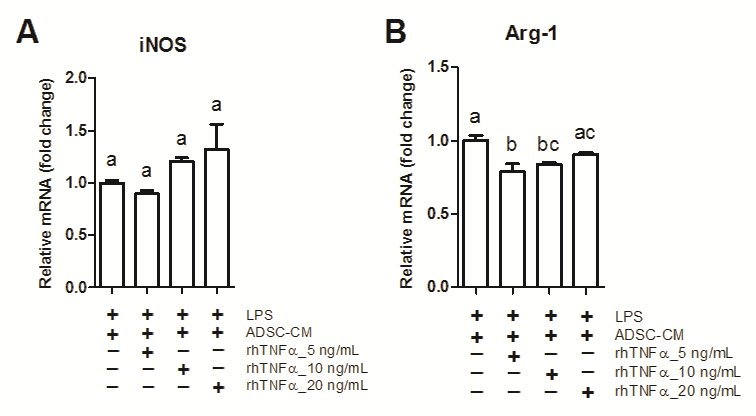
**

| **Table S1.** Primary antibodies used for immunohistochemical **and immunofluorescence** staining. | | | |
| --- | --- | --- | --- |
| **Primary antibody** | **Species** | **Working condition** | **Source** |
| N-Cadherin | Rat | 1:50 | Abcam |
| Cytokeratin 8/18/19 | Rat | 1:100 | Abcam |
| α-SMA | Rat | 1:100 | Abcam |
| iNOS | Rat | 1:100 | Abcam |
| ARG-1 | Rat | 1:100 | Cell Signaling |
| **IL-6** | **Rat** | **1:250** | **R&D** |
| **CD163** | **Rat** | **1:50** | **Abcam** |
| **TGF-β1** | **Rat** | **1:50** | **R&D** |
| Nucleoli | Human | 1:100 | Millipore |
| *Abbreviations: SMA, smooth muscle actin; iNOS, inducible nitric oxide synthase; ARG-1, Arginase-1**; IL-6, interleukin-6; TGF-β1, transforming growth factor-beta 1**. | | | |

**Table S2.** Sequences of RT-PCR primers for macrophage marker genes.

| **Gene** | **Sequences** |
| --- | --- |
| iNOS | gAgTgAggAgCAggTTgAgg**/**CCAAggTgTTgCCCTTTTT |
| Arg-1 | TTgATgTTgATggACTggAC**/**TCTCTggCTTATgATTACCTTC |
| β-actin | TgACAggATgCAgAAggAgA**/**TAgAgCCACCAATCCACACA |
